# Digital Image Analysis Using *FloCIA* Software for Ornamental Sunflower Ray Floret Color Evaluation

**DOI:** 10.3389/fpls.2020.584822

**Published:** 2020-11-09

**Authors:** Martina Zorić, Sandra Cvejić, Emina Mladenović, Siniša Jocić, Zdenka Babić, Ana Marjanović Jeromela, Dragana Miladinović

**Affiliations:** ^1^Institute of Lowland Forestry and Environment, University of Novi Sad, Novi Sad, Serbia; ^2^Sunflower Department, Institute of Field and Vegetable Crops, Novi Sad, Serbia; ^3^Faculty of Agriculture, University of Novi Sad, Novi Sad, Serbia; ^4^Faculty of Electrical Engineering, University of Banja Luka, Banja Luka, Bosnia and Herzegovina

**Keywords:** sunflower, breeding, ornamental value, digital UPOV, software, classification

## Abstract

As an esthetic trait, ray floret color has a high importance in the development of new sunflower genotypes and their market value. Standard methodology for the evaluation of sunflower ray florets is based on International Union for the Protection of New Varieties of Plants (UPOV) guidelines for sunflower. The major deficiency of this methodology is the necessity of high expertise from evaluators and its high subjectivity. To test the hypothesis that humans cannot distinguish colors equally, six commercial sunflower genotypes were evaluated by 100 agriculture experts, using UPOV guidelines. Moreover, the paper proposes a new methodology for sunflower ray floret color classification – digital UPOV (dUPOV), that relies on software image analysis but still leaves the final decision to the evaluator. For this purpose, we created a new *Flower Color Image Analysis* (*FloCIA*) software for sunflower ray floret digital image segmentation and automatic classification into one of the categories given by the UPOV guidelines. To assess the benefits and relevance of this method, accuracy of the newly developed software was studied by comparing 153 digital photographs of F_2_ genotypes with expert evaluator answers which were used as the ground truth. The *FloCIA* enabled visualizations of segmentation of ray floret images of sunflower genotypes used in the study, as well as two dominant color clusters, percentages of pixels belonging to each UPOV color category with graphical representation in the CIE (International Commission on Illumination) L^∗^a^∗^b^∗^ (or simply Lab) color space in relation to the mean vectors of the UPOV category. Precision (repeatability) of ray flower color determination was greater between dUPOV based expert color evaluation and software evaluation than between two UPOV based evaluations performed by the same expert. The accuracy of *FloCIA* software used for unsupervised (automatic) classification was 91.50% on the image dataset containing 153 photographs of F_2_ genotypes. In this case, the software and the experts had classified 140 out of 153 of images in the same color categories. This visual presentation can serve as a guideline for evaluators to determine the dominant color and to conclude if more than one significant color exists in the examined genotype.

## Introduction

Sunflower (*Helianthus annuus* L.), is a common plant species that is grown for different purposes. Following its introduction into Europe by Spanish sailors, the ‘flower of the sun’ or ‘the New World flower,’ as it was called at the time, quickly gained popularity as an ornamental plant (Kaya et al., 2012). For almost 200 years, sunflowers were grown in Europe exclusively as ornamental plants (Cvejić et al., 2016). Today, most of the cultivated sunflower plants are used for oil production, as confectionery sunflowers, or as bird feed. In recent years, sunflower has been “rediscovered” as an interesting ornamental plant due to its beauty and possibility to be used both as an ornamental cut flower and as potted plant (Mladenović et al., 2016). Since sunflower has moderate drought tolerance and can be grown in different agroecological conditions (Miladinović et al., 2019), its utilization, as ornamental plant, that is based upon floral and vegetative characteristics rather than seed production, permits its cultivation under even wider spectrum of agroecological conditions (Vuppalapti, 2005).

The main esthetic quality of ornamental sunflower is high variation in shape and texture, as well in the color of the flower. Sunflower inflorescence consists of fertile disk florets, located in the internal part of the flower head and circular arranged sterile ray florets around them. The color of sunflower disk florets can vary from dark red to completely white. Ray florets are mostly yellow, but may also appear in different shades of red, orange, lemon-yellow, white, or combination of these colors (Cvejić et al., 2016). New types of floricultural crops are in demand at the market and among consumers eager for new varieties with ornamental value, such as flowers with new shapes and colors (Shibata, 2008). According to Jocić et al. (2015), ornamental sunflower producers are, considering market requirements, mostly interested in the variation of flower color, and the structure of ray florets. As in most of the cultivated plants, the market also defines the breeding objectives in ornamental sunflower, where the focus is on desirable plant architecture, colors of ray and disk florets, and a prolonged duration of flowering (Cvejić and Jocić, 2010).

The quality of new plant genotypes can be evaluated using numerous criteria, but for ornamental plants, the most important criterion is flower color, hence extensive selection and breeding is done to develop new phenotypes (Bohorquez-Restrepo, 2015). The same stands for ornamental sunflower breeding, where the most important feature in terms of ornamental value is the color of ray florets (Divita et al., 2012). Phenotyping during creation of new genotypes is done using UPOV descriptor for sunflower (UPOV, 2000), and the complete process of phenotypic traits evaluation, including ray floret color determination, is done subjectively. Considering the fact that people see colors differently, the subjective approach to the description of crucial ornamental sunflower traits, such as ray floret color, could lead to inconsistency between how ray floret color is observed by breeders as opposed to catalog description of a commercial variety. This discrepancy could potentially have a negative influence on further research of new genotypes, ornamental sunflower market, and customer preferences, as well as protection of breeders’ rights.

In this paper, we have described a new methodology for ornamental sunflower ray floret color classification, based on in-house developed *Flower Color Image Analysis* (*FloCIA*) software performed in Lab color space. Based on the UPOV descriptor for sunflower, we have developed a new, digital UPOV for sunflower ray floret color classification (dUPOV), that relies on software image analysis but still leaves to evaluator the possibility of making the final decision.

## Materials and Methods

### Plant Material

Images of six proprietary ornamental sunflower genotypes were used for the evaluation of the ray floret colors, as well as segmentation of UPOV categories (Table 1), while images of 153 F_2_ genotypes were used for testing of *FloCIA* software accuracy^1^.

**TABLE 1:** Ray floret color evaluation of studied ornamental sunflower genotypes.

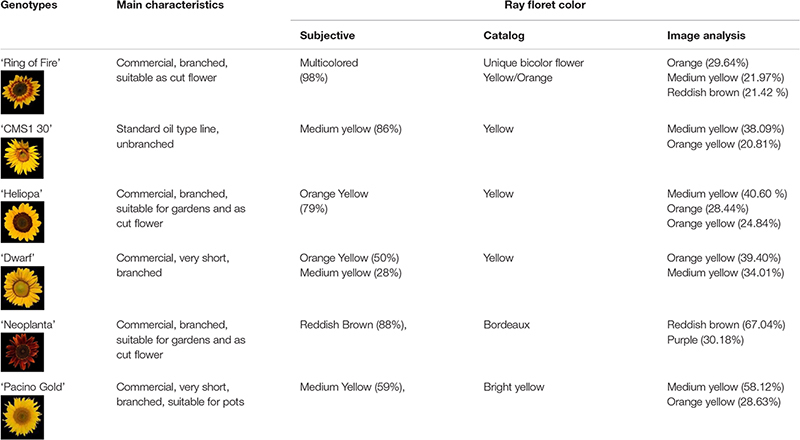

**Subjective* – evaluation done by 100 evaluators. Data presented in the percentages for values over 20%. *Catalog* – color description in commercial catalog. Image analysis – Color evaluation with the use of *FloCIA* software. Data presented in the percentages for values over 20%.*

### Experimental Trial

The material was collected during the 2017 and 2018 growing seasons, in Serbia (45.26°N, 19.83°E). Observations of ray floret color were carried out in the flowering phase F 3.2 as recommended by the UPOV guidelines for sunflower (UPOV, 2000). Visual assessment based on the UPOV guidelines was performed in the field. All examined genotypes were photographed using Nikon 3300 camera, with 6000 × 4000 resolution, in.png format. The photographs of each sunflower flower were taken without flash, during early morning with the sun facing the flowers to ensure environmental conditions as uniform as possible for each photograph taken. The white background (cloth) was placed behind each flower in order to facilitate background subtraction and ray floret segmentation. Ray floret color evaluation was performed using the data collected and analyzed from two separate studies; assessments based on the UPOV descriptor for sunflower (UPOV, 2000) and the proposed methodology based on in-house designed software for digital image processing for image segmentation, petals’ pixels classification into UPOV color groups and statistical analysis.

### Methodology Based on UPOV Guidelines

The UPOV descriptor for sunflower provides guidelines for the assessment of 11 characteristics related to ray and disk sunflower florets. Descriptor is supported by the *Description of Components and Varieties of Sunflower*, created by GEVES, the French Variety and Seed Study and Control Group in 2000, which provides a set of pictures to facilitate the calibration of scale notation for each characteristic. In the descriptor, color of ray florets has been defined as *yellowish white, light yellow, medium yellow, orange yellow, orange, purple, reddish brown*, and *multicolored*, and the set of photographs was provided (Table 2).

**TABLE 2:** Segmentation of the International Union for the Protection of New Varieties of Plants (UPOV) color categories with *Flower Color Image Analysis* (*FloCIA*) and color clouds obtained by analysis with HSV (H-hue, S-saturation and V-value of light intensity) and Lab color space.

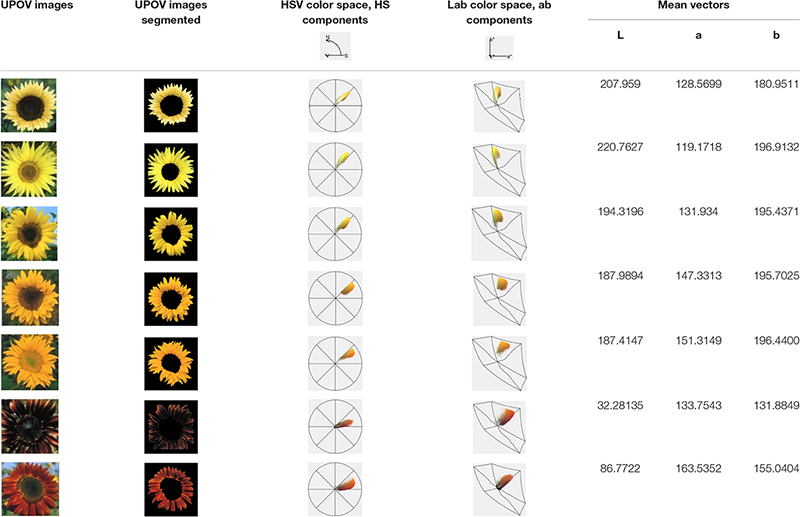

*UPOV images – Color groups based on UPOV descriptor for sunflower. From top to the bottom of the column: yellowish white, light yellow, medium yellow, orange yellow, orange, purple, reddish brown.*

Evaluation of the ray floret colors of the examined ornamental sunflower genotypes was done by 100 people with the use of the UPOV guidelines. The evaluators were mainly employees in the Institute of Field and Vegetable Crops or professors and students at different levels of horticulture studies at the Faculty of Agriculture, University of Novi Sad. During 2018, the evaluators were given a questionnaire containing photographs from UPOV descriptor for each ray floret color category and photographs of examined sunflower genotypes (Table 1, column Genotypes, rows A–F). Before the assessment, the evaluation process has been thoroughly explained to each evaluator. The evaluators were asked to define one or more colors present in ray florets of examined sunflower genotypes, based on the provided UPOV guidelines.

### Methodology Based on FloCIA Software

*Flower Color Image Analysis* (*FloCIA*) software was used for segmentation of the UPOV categories. Segmentation included separation of flowers from the background and removing disk florets to get only ray florets. After the segmentation, color clouds in HSV (H-hue, S-saturation and V-value of light intensity) and Lab color space were analyzed (Table 2). In HSV color model V component was omitted (2D presentation) for better visualization of colors., and all the pixels with hue outside the range of 17.99-66.95 (yellow and red color area) and with saturation lower than 12.5% of the maximum saturation were removed.

**FIGURE 1:**
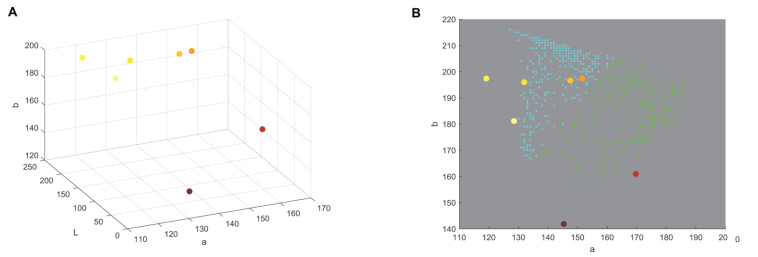
Determined mean vectors for International Union for the Protection of New Varieties of Plants (UPOV) color categories in **(A)** 3D Lab color space and **(B)** 2D ab color space.

For the purpose of classification into one of seven UPOV classes (UPOV 1 - UPOV 7) in Lab color space, mean vectors of UPOV categories of sunflower ray florets have been calculated. Lab color space is used when is necessary to determine color differences, and where each pixel of the image is written with three values: L^∗^, a^∗^, b^∗^. Pixels are linked to the UPOV category by the nearest neighbor method (Altman, 1992), where Euclidean distance was calculated for each pixel from all seven mean vectors, and the pixels were classified into the category for which the distance is the shortest. Value of each category was calculated by counting the pixels from the same category of the test sunflower and dividing them by the total number of pixels of the segmented test sunflower ray florets. The mean vector numerical values are given in the Table 2. The centers of the cluster (middle vectors) of the UPOV category (seven larger dots) were visually presented in 3D representation (Figure 1A), and in 2D color distribution (Figure 1B).

Finally, software *FloCIA* was used for segmentation and classification of the six examined genotypes. The basic techniques used for this purpose were smoothing and standard deviation filtering and color and texture segmentation. Clustering of ray florets included their grouping into three clusters by using the k-means algorithm. Visually, images of two dominant clusters (dominant colors) were shown in the form of images of sunflowers, with separate colors. Additionally, the pixel layout was displayed in the Lab color space (blue dot in the 3D image Lab color space). The closest UPOV category to blue dot shows dominant color of the examined sunflower.

The accuracy of the software was tested based on the results of segmentation and colors classification of 153 images of F_2_ ornamental sunflower genotypes and compared with the answers of an expert in two replications. The first replication was on a day of evaluation and the second one after 7 days.

The data and code that support the findings of this study are publicly available on https://zenodo.org/record/4068475#.X31utGgza71.

### Data Analysis

Reliability coefficients were calculated in order to assess the reliability of UPOV methodology for sunflower ray floret color evaluation. Result of all evaluators’ answers were processed and analyzed statistically in the R environment (R Development Core Team, 2011), with the use of Fleiss Kappa statistical model (Fleiss, 1971). This model is commonly used to evaluate the validity of multiple raters which are classifying a subject in multiple categories based on kappa (k) value. Considering that possible values of kappa are in range from −1 to 1, basic opinion is that if value of kappa is closer to 1, the agreement among raters is stronger, the validity of results is higher, and vice-versa, if the value of k is closer to −1, the agreement among raters is weaker and the validity of the obtained results is lower. According to Xie et al. (2017), Kappa values in ranges of (0–0.2), (0.2–0.4), (0.4–0.6), (0.6–0.8), (0.8–1) are an indication of slight, fair, moderate, substantial and almost perfect consistency, respectively.

Based on 100 evaluator scores of ray floret color of six tested sunflower genotypes, the percentages of evaluators who associated ray floret color to any of the UPOV color categories was correlated with the percentages of pixels in the corresponding UPOV color categories for all genotypes.

Objectivity of the *FloCIA* software, as well as its accuracy and precision (repeatability) were estimated using matching and confusion matrix (Visa et al., 2011). Expert annotations were used as the ground truth (evaluator’s scores). They were made after the evaluator was presented with original sunflower images followed by images of two clusters of dominant colors, percentages of pixels belonging to individual UPOV categories and pixel distribution in the ab plane of the Lab color space, and the positions of the mean vectors of examined sunflowers in Lab color space in relation to the mean vectors of the UPOV categories. Two scenarios were examined. In the first one (matching matrix), the *FloCIA* was used as a tool in assisted decision-making methodology, to show subjectivity of evaluator due to a high number of photos and the time distance. In this scenario, the algorithm was applied to match the results of the same evaluator on 153 photographs of F_2_ sunflower genotypes using traditional UPOV method for sunflower ray floret color classification in two replications. The first replication was marked as UPOV_EP1 and the second one, comprising classifications of the same evaluators after 7 days, as UPOV_EP2. The second scenario (confusion matrix) was created to visualize matching of an expert evaluator result using the *FloCIA* software assistance as a ground truth, and automatic sunflower color classification. Data in the *FloCIA assisted* rows were presented as real (*actual*) color categories in which the analyzed genotypes were classified (ground truth), and the data belonging to the *Automatic classification* were determined by an algorithm (*predicted*). The data gave information on how many sunflower genotypes were classified in an appropriate color category that match ground truth data, and how many were mistakenly automatically classified in another color category by an algorithm. From this confusion matrix, the accuracy of the automatic classification and precision (repeatability) for each UPOV category was calculated. Accuracy was calculated as the ratio of the number of correct classifications (sum of all diagonal values) to the total number of classifications. Precision was calculated in the same way that was used for the matching matrix.

## Results

### Color Evaluation by UPOV Guideline

Based on the evaluators’ answers by UPOV guidelines, genotypes ‘CMS1 30’ and ‘Neoplanta’ had a high percentage of the same answers (more than 80%) (Table 1). ‘Neoplanta’ was classified as *reddish brown* (88% respondents), although some of the answers suggest more than one color (11%), while ‘CMS1 30’ was classified as *medium yellow* (86%). Based on the highest answer percentage, both ‘Heliopa’ and ‘Dwarf’ were classified as *orange yellow* with 79% and 50% of answers, respectively. With the highest percentage of 59%, ‘Pacino Gold’ was classified as *medium yellow*. Sunflower genotype ‘Ring of Fire’ was classified as *multicolored*, with 98% of evaluators matched it with this category. Considering that the *multicolored* color category in UPOV guidelines was not clearly defined, evaluators were asked during the assessment if they can determine two or more colors of this genotype based on UPOV color guidelines. Out of 100 evaluators, 77% reported that they were able to determine two or more colors, but only 3.96% of them observed three or more colors. Most of the evaluators reported that this multicolored sunflower variety could be described by two dominant color categories: *medium yellow* and *reddish brown*. In order to examine the validity of the test, the agreement among evaluators has been calculated, as k- value. Based on this value and strength of agreement, the obtained results can be described as moderate agreement (k = 0.542).

### Digital Image Analysis (dUPOV) Using FloCIA Software

**FIGURE 2:**
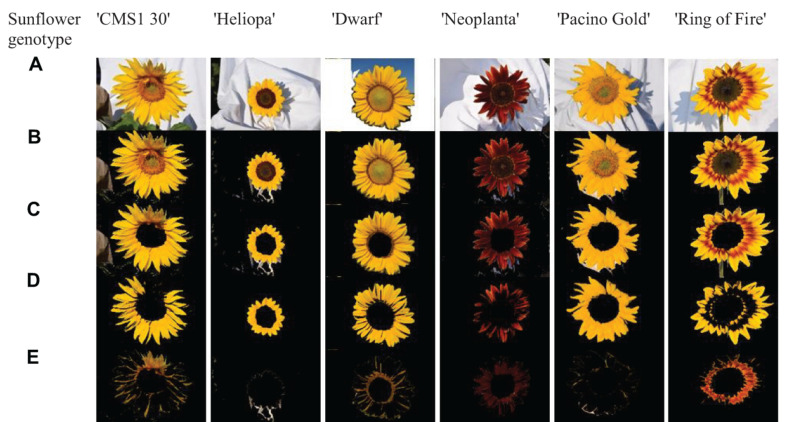
The images of six examined sunflower (H. annus) genotypes (‘CMS1 30,’ ‘Heliopa,’ ‘Dwarf,’ ‘Neoplanta,’ ‘Pacino Gold,’ and ‘Ring of Fire’) were segmented (excluded background and disk florets) and clustered using the k-means algorithm: **(A)** original images, **(B)** color segmentation, **(C)** texture segmentation, **(D,E)** two dominant clusters.

In the process of color segmentation, ray and disk florets were extracted from the background. Disk florets of investigated sunflower genotypes were mostly characterized by different shades of similar colors as their ray florets, but the texture was different. The difference among textures of ray and disk florets allowed design of software for image segmentation and removal of sunflower disk florets in test images. Image segmentation was conducted by designing a texture segmentation algorithm based on histogram equalization, standard deviation filtering, and morphology operation. Step by step segmentation of parental line sunflower’s images were shown in Figures 2B,C. A white cloth was used behind sunflowers during image recording and no manual correction was used (Figure 2A). After segmentation, k-Means clustering in Lab color space was used to determine the dominant ray floret color clusters. Lab color space was perceptually uniform to the human color vision, meaning that the same amount of numerical change in these values corresponds to about the same amount of the visually perceived change. Luminance component (L) was neglected so as to avoid unfavorable ambient illumination conditions. The results are given in Figures 2D,E.

Finally, ray florets of six tested genotypes were segmented, and pixels of ray florets of each genotype were clustered into two dominant colors. Visualization of this process was presented in Figures 2C–E. The columns from left to right show segmented ray florets, pixels belonging to the first dominant color cluster, and pixels belonging to the second dominant color cluster.

**FIGURE 3:**
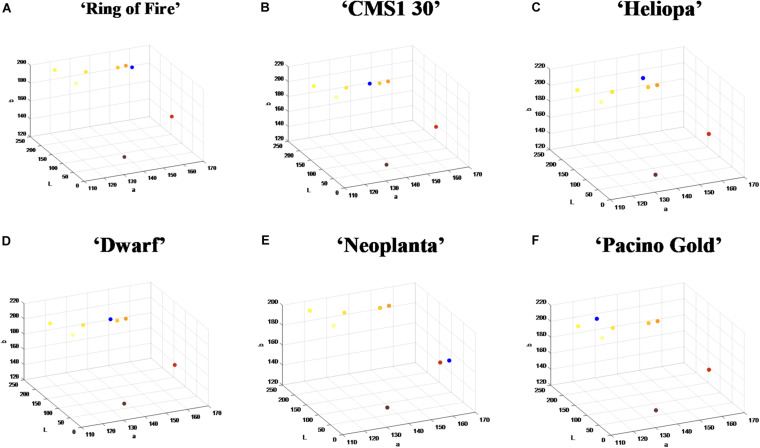
The position of dominant color clusters for each examined genotype. Dots in yellowish white, light yellow, medium yellow, orange yellow, orange, purple, and reddish brown represent one of the seven International Union for the Protection of New Varieties of Plants (UPOV) color categories. The dominant color in tested genotypes is marked with blue dot. **(A–F)** Represent each tested sunflower genotype.

The nearest neighborhood methodology of classification was used thereafter, so that Euclidean distances from the mean vectors of UPOV color categories were calculated for each pixel from the two dominant clusters, and pixels were classified using the smallest Euclidean distance. Visualization of this process for six commercial sunflower genotypes was presented in Figures 3A–F. Along with visual representation of mean vectors of the seven UPOV color categories (dots in *yellowish white*, *light yellow*, *medium yellow*, *orange yellow*, *orange*, *purple*, and *reddish brown*), that were obtained by processing the original UPOV images, a dominant color in tested genotypes was marked with blue dots. This process guides the visual determination of sunflower ray florets color category: by comparing the distances of blue dot from the dots of UPOV colors in the Lab color space, it becomes clearer which of the seven UPOV colors were dominant in a tested genotype (Figures 3A–F).

**FIGURE 4:**
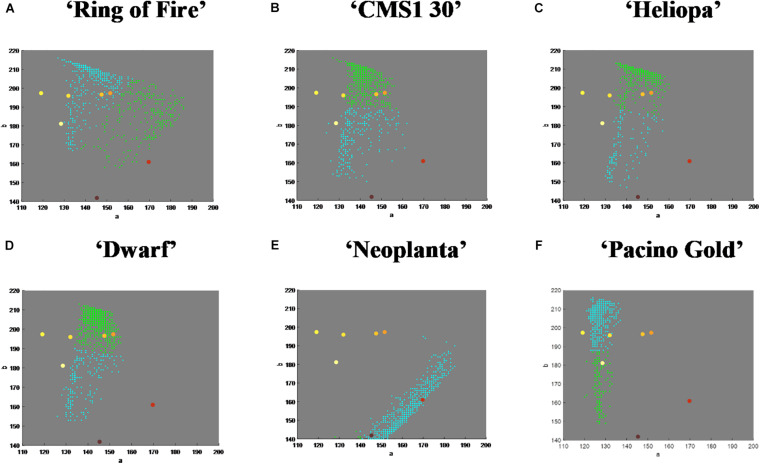
The amount and distribution of pixels of each International Union for the Protection of New Varieties of Plants (UPOV) color category in Lab color space. Dots in yellowish white, light yellow, medium yellow, orange yellow, orange, purple, and reddish brown represent one of the seven UPOV color categories. The dominant color in tested genotypes is marked with blue dots. Pixels belonging to two dominant color clusters in tested genotypes in ab color space are roughly represented with blue and green dots. **(A–F)** Represent each tested sunflower genotype.

Additional visualization of mean vectors of clusters showed distribution and number of pixels in chrominance “ab” plane of Lab color space (Figures 4A–F). For example, genotype ‘Ring of Fire’ presented disperse pixel layout, showing presents of all UPOV color categories, while ‘Neoplanta’ presented narrow distribution of pixels focused around *purple* and *reddish brown* UPOV color categories.

Quantification of the main color of ray florets of the six examined sunflower genotypes was performed using Lab color space. The mean vectors of sunflower ray florets have been calculated (Table 3). The final results of image analysis were presented in the percentage of pixels per each UPOV color category (Table 1). Finally, the percentage of pixels per UPOV category and visual representation of two dominant colors and their mean vectors are used by humans in the decision-making process.

**TABLE 3:** Quantification of the main color of ray florets of the six examined sunflower genotypes with mean vectors obtained with Lab color space.

Genotype	L	a	b
‘Ring of Fire’	169.0986	152.4655	197.8293
‘CMS1 30’	179.3181	141.6079	200.0990
‘Heliopa’	195.4105	145.8504	205.7754
‘Dwarf’	181.1503	143.4153	200.9932
‘Neoplanta’	68.3184	165.5987	159.9250
‘Pacino Gold’	184.7126	139.0641	201.5160

**L* – lightness; *a* – component which varies from green to red; *b* – component which varies from blue to yellow.*

### Correlation Between UPOV and dUPOV Values

**FIGURE 5:**
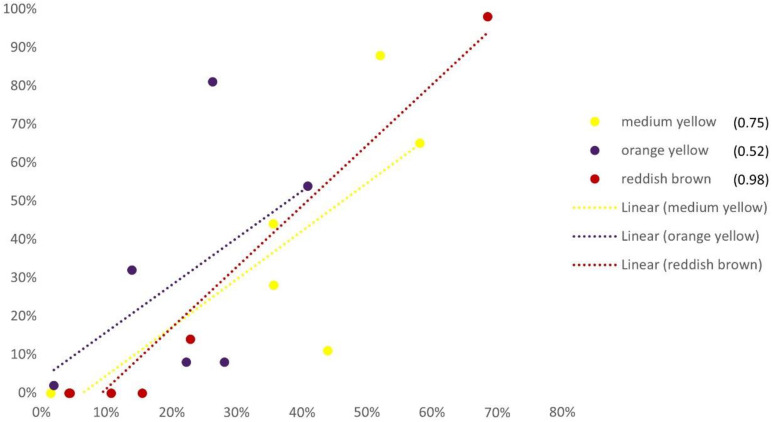
Correlation among evaluators’ score and number of pixels of each International Union for the Protection of New Varieties of Plants (UPOV) color category of six examined sunflower genotypes (‘Neoplanta,’ ‘Heliopa,’ ‘CMS1 30,’ ‘Ring of Fire,’ ‘Pacino Gold,’ and ‘Dwarf’). X-axis represents the percentage of pixels for all genotypes; y-axis represents the percentage of evaluators scores of all genotypes.

The correlation between evaluator scores and number of pixels follows the trend line (Figure 5) for only three color categories (*medium yellow*, *orange yellow* and *reddish brown*). Other colors did not have a sufficient number of pixels and therefore were not recognized by evaluators.

### FloCIA Software Accuracy Testing

*FloCIA* software’s accuracy and repeatability has been tested on the image set containing 153 photographs of F_2_ ornamental sunflower genotypes.

In order to examine the objectivity of the software, its accuracy and precision (repeatability) were calculated using the confusion matrix. To assess if the software is more accurate and precise than the human evaluation, the same parameters were calculated using the matching matrix on expert color evaluation based on the traditional UPOV guidelines. The results of the matching matrix (Table 4) and confusion matrix (Table 5) presented a visualization of accuracy and precision (repeatability) of these two processes of sunflower color evaluation.

**TABLE 4:** Matching matrix of expert answers on the dominant color classification by the International Union for the Protection of New Varieties of Plants (UPOV) guidelines in two replications (UPOV_EP1 and UPOV_EP2), 7 days apart.

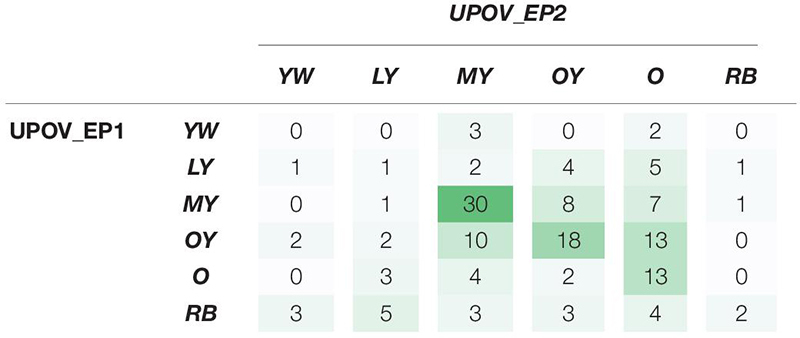

*The values on the diagonal of the matching matrix indicate the number of the same evaluations, while the values outside the diagonal indicate the number of evaluations that differ in two replications. Color categories from UPOV guidelines: YW – yellowish white, LY – light yellow, MY – medium yellow, OY – orange yellow, O – orange, RB – reddish brown.*

**TABLE 5:** Confusion matrix of evaluator answers obtained with the assistance of *FloCIA* software and automatic sunflower color category classification.

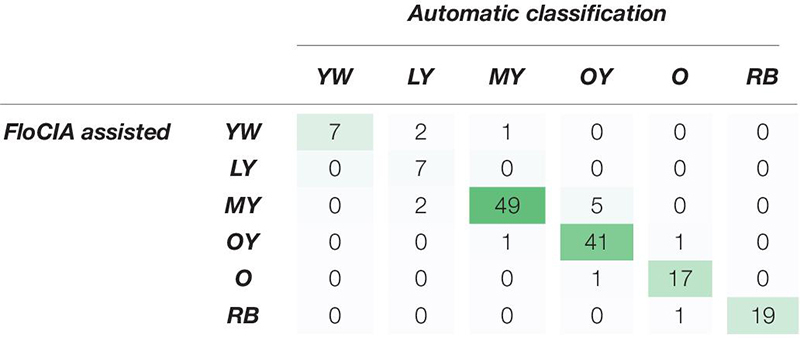

*The data in *FloCIA assisted* rows present real (*actual*) color categories that analyzed genotypes were classified in (ground truth), and the data belonging to the *Automatic classification* were determined by an algorithm (*predicted*). The values on the diagonal of the confusion matrix indicate the number of evaluations that were the same for *FloCIA* assisted color evaluation (ground truth) and automatic evaluation, and the values outside the diagonal indicate the number of evaluations that differ. Color categories from the International Union for the Protection of New Varieties of Plants (UPOV) guidelines: YW – yellowish white, LY – light yellow, MY – medium yellow, OY – orange yellow, O – orange, RB – reddish brown.*

The matching matrix was used to visualize the matching of the results of two replications: the first UPOV_EP1 and the second after 7 days UPOV_EP2 (Table 4). The values on the diagonal of the matching matrix indicate the number of the same evaluations (0,1,30,18,13,2), while the values outside the diagonal indicate the number of evaluations that differ in two replications, carried out by the same expert evaluator using the traditional UPOV color evaluation method. The matching matrix visualization mostly showed scattered answers given by the experts, whereas the highest number of replications was characteristic of the *medium yellow* (MY) color group (30) and *orange yellow* (OY) color group (18), as confirmed by the calculated values of precision 57.69%, 51.43%, respectively (Table 6). The calculated accuracy of single expert evaluator using the traditional UPOV method in two replications seven days apart was 41.83%.

**TABLE 6:** Calculated precision for each International Union for the Protection of New Varieties of Plants (UPOV) color category found as a dominant color of tested sunflower genotypes.

Precision of matching color categories	YW *yellowish white*	LY *light yellow*	MY *medium yellow*	OY *orange yellow*	O *orange*	RB *reddish brown*
Expert evaluator in two replications by UPOV guidelines	0.00%	8.33%	57.69%	51.43%	29.55%	50.00%
Expert evaluator supported by *FloCIA* software and automatic classification	100.00%	63.64%	96.08%	89.13%	89.47%	100.00%

The confusion matrix (Table 5) was created to visualize the matching of the data in *FloCIA assisted* methodology. The rows present real (*actual*) color categories that analyzed genotypes were classified in (ground truth), and the data belonging to the *Automatic classification* were determined by an algorithm (*predicted*). The values on the diagonal of the confusion matrix indicate the number of evaluations that were the same for *FloCIA* assisted color evaluation (ground truth) and automatic evaluation (7,7,49,41,17,19), and the values outside the diagonal indicate the number of evaluations that differ. The calculated accuracy of automatic sunflower color classification was 91.50%, which was supported by the high precision of matching color categories above 60% (Table 6).

## Discussion

Accurate phenotype classification is one of the requirements of the ornamental plant market, which is becoming increasingly more sophisticated and demanding (Guerra Mattos et al., 2020). The same stands for ornamental sunflower breeding and marketing which is expected to adopt automatic classification, in order to improve breeding efficiency and quality of products offered to consumers (Lino et al., 2011). Color determination is highly subjective, and can possibly even lead to misunderstandings, wrong genotype descriptions and unsatisfied producers. Digital imaging in combination with efficient analytic software could be a powerful tool efficient and accurate transformation of qualitative measurements of phenotypic traits like color and shape into quantitative data (Bohorquez-Restrepo, 2015).

In this paper we describe the first attempt to use digital image analysis for ray floret color evaluation in ornamental sunflower. Based on image analysis algorithms, in-house created software, *FloCIA* automatically detected dominant colors of ray florets in six ornamental sunflower genotypes, thus offering novel methodology for objective and more precise ornamental sunflower phenotyping. In ornamental plants, digital image technology has been used for gerbera flower classification (Lino et al., 2011), rose shape analysis (Miao et al., 2006), bedding plant species quality assessment (Parsons et al., 2009), flower color pattern determination in *Primula sieboldii* E. Morren (Yoshioka et al., 2004), as well as evaluation of *Anthurium* ‘Tropical’ postharvest quality (Guerra Mattos et al., 2020). In sunflower, image analysis so far has been used for early detection of broomrape infection (Cochavi et al., 2017; Ortiz-Bustos et al., 2017; Lati et al., 2019), weed mapping (López-Granados et al., 2016), architecture-based organ segmentation (Gélard et al., 2016) and floral dimension determination (Sunoj et al., 2018). Common for all image analysis studies is that they can either replace the currently used parameters or provide additional characteristics with good discriminating power, determined in an objective and standardized way (Lootens et al., 2013).

Our study proposes using dUPOV to eliminate shortcomings of the UPOV-based methodology, such as subjectivity and inability of evaluators to differentiate colors, as well as the quality of photographs in the UPOV guidelines. For example, evaluators could not say with the certainty if some genotypes, such as ‘Neoplanta,’ should be described as single colored or multi-colored, while being certain that ‘Ring of Fire’ could be described as multicolored. Although digital image results also showed this color as the dominant, the *FloCIA* software recognized the presence of *purple* in this genotype which covers 30.18% of its color (Table 1). Results for the other tested genotypes, such are ‘CMS1 30’ and ‘Heliopa,’ showed the dominance of one color - for ‘CMS1 30’ *medium yellow* with 86%, and for ‘Heliopa’ *orange yellow* with 79%. In both cases only 6% of the evaluators observed the presence of the second dominant color which, according to the results of the *FloCIA* software, was *orange yellow* (20.81%) for ‘CMS1 30’ and *orange* (28.44%) for ‘Heliopa.’ Genotype ‘Pacino Gold’ seemed to be easy for color evaluation. However, the presence of different shades of yellow and orange misguided the evaluators, but its color was clearly identified by the *FloCIA* software. Results of image analysis of this genotype showed the dominance of *medium yellow* (58.12%), but also the presence of *orange yellow* (28.63%) which evaluators failed to observe. Most of the evaluators defined ‘Ring of Fire’ as a *multicolored*, with *reddish brown* and *medium yellow* as two dominant colors. Based on the results of image analysis using *FloCIA* software, this genotype was characterized with three colors: *orange*, *medium yellow, and reddish brown*, with percentages of 29.64, 21.97, and 21.42, respectively. Statistical analysis confirmed the uncertainty of evaluators with moderate value of kappa for the agreement among evaluators. Similar to the results of our study, Kendal et al. (2013) found that analysis of digital images of plants of eleven grassland species taken with digital cameras was a practicable method for quantifying color and estimating color difference between flowers both in the field and in the laboratory. The same stands for Garcia et al. (2014), who used digital imaging to map flower colors and concluded that this method presents a significant new opportunity to reliably map flower colors.

A quantitative and objective measure of plant color has the potential to improve plant management through improved estimation of plant dynamics and improved modeling of human-plant interactions (Kendal et al., 2013). Digital images can provide information on the color patterns of objects; this information is composed of pixels, their locations and color depths (Yoshioka et al., 2004). Based on image analysis algorithms, we have created the *FloCIA* software that automatically classifies image pixels and gives guidelines to a person who makes the final conclusion. Using *FloCIA*, none of the tested genotypes can be described as single colored, while visual estimation placed all six tested genotypes into one dominant color. Although the base color of ray florets was visually scored, the *FloCIA* enabled the detection of different shades of the same color. In the case of sunflower, using digital image, we could distinguish hues of yellow, orange and red color. For example, genotype ‘CMS1 30’ had predominant *medium yellow* (38.09%) but all other colors from the UPOV guideline we were also detected by *FloCIA* software. We have also tested the accuracy of the *FloCIA* software trough matching and confusion matrixes. The results showed that there is scattering of the data obtained by evaluations based on UPOV guidelines, compared to *FloCIA* software automatic color classification. Both calculated parameters, accuracy and precision (repeatability) of matching of each color category, were unequivocally higher with the use of *FloCIA* software. Singh et al. (2011) also pointed out the advantages of the use of image analysis in the flower color study, as it enables increased precision in estimation of variation by differentiating color groups into sub-groups, which in turn allowed for determination of variations within the sub-groups in a reliable manner. This fine color determination opens up new possibilities in ornamental sunflower breeding, production, marketing and trade, since Behe et al. (1999) in their study of consumer preferences revealed that flower color, followed by leaf variation, and the price, was the most important factor affecting consumer’s decision to buy certain ornamental variety and has direct influence on its commercial value. Hence developing precise flower color phenotyping methods for color analysis is essential for providing accurate, quantitative information useful both in breeding and variety marketing and commercialization.

## Conclusion

The main advantages of digital image analysis methods are their accuracy as well as clear and concise information for color evaluation in a breeding process. The proposed digital image (dUPOV) methodology using *FloCIA* software for sunflower ray floret color evaluation offers objective information supported with quantitative data regarding the dominant color category of ray florets, making the final evaluations made either by breeders or traders more accurate and carried out with more confidence. dUPOV groups the pixels of segmented ray florets into two dominant clusters and graphically presents the position of their mean vectors in Lab color space, thus providing the breeders with objective information on ray floret color of the newly created varieties. Since color patterning contributes to important plant traits that influence ecological interactions, ornamental plants breeding, and agricultural performance, the proposed dUPOV image analysis methodology could be adapted and applied for similar tasks in different fields of horticulture, agriculture and plant research.

## Data Availability Statement

The raw data supporting the conclusions of this article will be made available by the authors, without undue reservation.

## Ethics Statement

The requirement for ethics approval for this study was waived by Ethics Committee Board of the Faculty of Agriculture, University of Novi Sad. All participants provided their written informed consent.

## Author Contributions

MZ, SC, and EM performed conceptualization. MZ and ZB performed methodology. MZ, SC, EM, and ZB performed formal analysis and investigation. MZ prepared original draft. SC, EM, and DM wrote, reviewed, and edited the manuscript. AM and DM acquired funding. SC and SJ performed resources. EM and SC performed supervision. All authors contributed to the article and approved the submitted version.

## Conflict of Interest

The authors declare that the research was conducted in the absence of any commercial or financial relationships that could be construed as a potential conflict of interest.

## Footnotes

1 https://zenodo.org/record/4068475#.X31utGgza71
